# Characterization of Mesenchymal Stem Cells Derived from Bisphosphonate-Related Osteonecrosis of the Jaw Patients’ Gingiva

**DOI:** 10.1007/s12015-021-10241-8

**Published:** 2021-09-22

**Authors:** Mengyu Li, Jiajia Wang, Yejia Yu, Yuqiong Zhou, Yueqi Shi, Wenjie Zhang, Geehun Son, Jing Ge, Jun Zhao, Chi Yang, Shaoyi Wang

**Affiliations:** 1 Department of Oral Surgery, Shanghai Engineering Research Centre of Advanced Dental Technology and Materials, Shanghai Key Laboratory of Stomatology & Shanghai Research Institute of Stomatology, National Clinical Research Centre for Oral Diseases, Ninth People’s Hospital, Shanghai Jiao Tong University School of Medicine, Shanghai, China; 2 Department of Oral Surgery, Shanghai Engineering Research Centre of Advanced Dental Technology and Materials, Shanghai Key Laboratory of Stomatology & Shanghai Research Institute of Stomatology, National Clinical Research Centre for Oral Diseases, Ninth People’s Hospital, Shanghai Jiao Tong University School of Medicine, Shanghai, China; 3 Department of Prosthodontics, Shanghai Engineering Research Centre of Advanced Dental Technology and Materials, Shanghai Key Laboratory of Stomatology & Shanghai Research Institute of Stomatology, National Clinical Research Centre for Oral Diseases, Ninth People’s Hospital, Shanghai Jiao Tong University School of Medicine, Shanghai, China; 4 Department of Oral Surgery, Shanghai Key Laboratory of Stomatology & Shanghai Research Institute of Stomatology, National Clinical Research Centre for Oral Diseases, Ninth People’s Hospital, Shanghai Jiao Tong University School of Medicine, Shanghai, China; 5 Department of Orthodontics, Shanghai Key Laboratory of Stomatology & Shanghai Research Institute of Stomatology, National Clinical Research Centre for Oral Diseases, Ninth People’s Hospital, Shanghai Jiao Tong University School of Medicine, Shanghai, China

**Keywords:** Bisphosphonates, Osteonecrosis, Gingival mesenchymal stem cells, Transplantation, Wound healing, Microenvironment, Oral mucosa

## Abstract

Bisphosphonate-related osteonecrosis of the jaw (BRONJ) is a clinical condition that specifically occurs in the oral cavity, characterized by retarded wound healing in oral mucosa accelerating the exposure of bone. Moreover, the pathological mechanism remains poorly understood. Gingival mesenchymal stem cells (GMSCs) play a critical role in gingival healing and soft tissue regeneration. Although previous studies have showed that bisphosphonates (BPs) are highly toxic to healthy GMSC, there is overall lack of direct evidence demonstrating the characterization of GMSCs derived from BRONJ patients. In present study, we isolated GMSCs for the first time from the central area of BRONJ patients’ gingiva (center-BRONJ GMSCs) and the peripheral area (peri-BRONJ GMSCs), and found that they exhibited decreased proliferation, adhesion, migration capacities and underwent early apoptosis in vitro compared control GMSCs. Notably, the central and peripheral BRONJ GMSCs transplantation in a mice excisional skin model also displayed lower cell survival rate and poor healing effects than that of controls. Mechanistically, TGF-β1 signaling pathway was suppressed not only in BRONJ patients’ gingival lesions but also in BRONJ GMSCs transplantation animal model. The results above suggested that under the microenvironment of BRONJ patients, the dysfunction of GMSCs and the suppressed TGF-β1 signaling pathway may be the vital factors in impaired gingival healing, thus contributing to persistent exposure of underlying bone and development of BRONJ. This study provides new insights into the prevention for BRONJ by improving the functions of GMSCs and upregulating TGF-β1 in accelerating gingival wound healing.

**Graphical Abstract:**

Schematic illustration of the dysfunction of BRONJ GMSCs in vitro and BRONJ GMSCs transplantation in a mice skin model delaying cutaneous wound healing mainly via suppressing TGF-β1 signaling pathway.

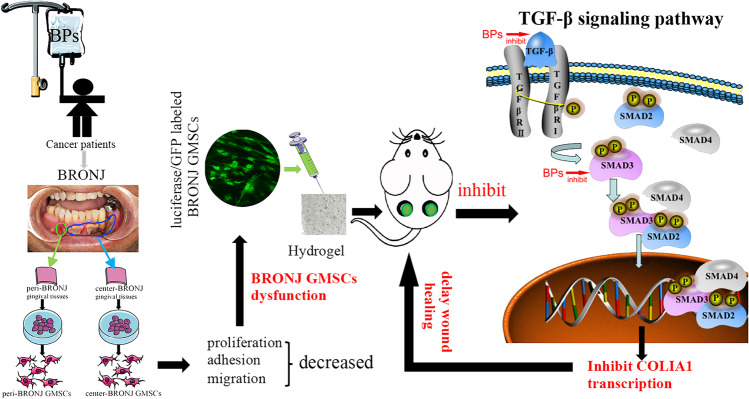

**Supplementary Information:**

The online version contains supplementary material available at 10.1007/s12015-021-10241-8.

## Background

Bisphosphonate-related osteonecrosis of the jaw (BRONJ) is a detrimental side effect that specifically occurs in the oral cavity in cancer patients receiving high doses of intravenous bisphosphonates (BPs), severely affecting patients’ quality of life [1]. The hallmark of BRONJ are necrotic bone exposure and retarded gingival healing, however, it is unclear why such the lesion should present with soft tissue defects as the primary clinical feature [2]. Furthermore, it is not known whether the BRONJ lesion initiates in the underlying bone or the superficial soft tissue.

Previously, we observed that BRONJ patients displayed cracked, edematous periodontal ligament fibers [3]. In the present study, we further found that BRONJ patients’ gingiva also appeared as disordered lamina propria and displayed notably depressed expressions of collagen. While numerous reports have been focusing on origin from the bone regarding the pathogenesis of BRONJ [4–7], our clinical observation that the progressive enlargement of gingiva defects in most BRONJ patients has inspired us to begin to investigate the microenvironment of oral mucosa as well as its role in the development of BRONJ. In comparison to other parts of the human body, the gingiva represent a unique oral tissue that directly links with the underlying bone and external bacterial environment, which is easily damaged by jaw trauma, especially tooth extraction and alveolar surgery [8]. In clinical, approximately 80% of BRONJ cases were typically localized to areas that were easily injured with very thin overlying mucosa due to the invasive dental procedures [9]. Recent research also suggested that poor gingival wound healing after tooth extraction in patients treated with BPs increases the susceptibility to bacterial infection, contributing to the development of BRONJ [10, 11]. Therefore, it is extremely urgent to explore the potential mechanism of impaired gingival healing and look for new therapeutic methods to promote wound healing in the prevention of BRONJ. Appropriate gingival wound healing is a complex, multistep process which largely relies on the functions of gingival mesenchymal stem cells (GMSCs): migration, proliferation, and differentiation [12, 13]. Extensive research has demonstrated that BPs are highly toxic to GMSCs in vitro [9, 14–16], however, there is a lack of direct evidence demonstrating the phenotypes and functions of GMSCs in BRONJ patients.

In this study, to embark on exploring the characterization of GMSCs derived from BRONJ patients’ gingiva and the exact mechanism of impaired gingival healing, firstly, we isolated GMSCs from the central lesions (center-BRONJ GMSCs) and the peripheral area of BRONJ patients’ gingiva (peri-BRONJ GMSCs) respectively. After that, we compared their proliferation, adhesion, migration, apoptosis in vitro with healthy GMSCs and their wound healing capacity in a mice excisional skin model. Secondly, we performed microarray analysis, real-time polymerase chain reaction (RT-PCR), western blot, and immunohistochemical (IH) staining to explore the exact mechanism. We anticipate the study will generate fresh insight into the pathogenesis of BRONJ and suggest the important ramifications in the prevention of BRONJ.

## Methods and Materials

### Sample Collection

Five BRONJ patients, ages 54–81 years, underwent surgery at the Department of Oral Surgery, Ninth Peoples Hospital. Debridement of the affected bone and gingiva was extended to reach healthy-appearing tissues [17, 18]. BRONJ patients were considered eligible for this study if they had a histologically confirmed advanced solid cancer and radiographic confirmation of bone metastases, receiving intravenous BPs and presented with exposed necrotic bone in the maxillofacial region at least eight. Patients were considered ineligible when they had received any radiotherapy, chemotherapy, immunotherapy, or hormonotherapy before the study. The control group included five healthy patients, older than 50, without wound healing-related diseases and no history of BPs use. They underwent third molar extraction, meanwhile, gingival tissues surrounding the tooth were collected. All patients provided written informed consent to participate in this study. Detailed patient information is listed in Supplement 1.

### Histological Analysis

Gingival samples fixed in 4% paraformaldehyde were embedded in paraffin and sliced for histological evaluation. Paraffin sections were stained with hematoxylin and eosin (H&E) as well as Masson staining. For IH staining, after deparaffinization, rehydration, antigen retrieval, permeabilization and blocking non-specific binding, sections were incubated in primary antibodies against collagen type I A1 (COLIA1) (Abcam; 1:200), transforming growth factor beta 1 (TGF-β1) (Abcam; 1:300), caspase 3 (Abcam; 1:200) at 4℃ overnight and secondary antibodies (Servicebio; 1:500) for 1 h at room temperature. DAPI (Abcam) at 1:500 was used as nuclear counterstain. Results were detected by fluorescence microscope (Olympus). BRONJ patients’ gingival samples were also assessed by TUNEL staining with Cell Death Fluorescein Detection Kit (Roche) following the manufacturer’s instructions. The experimental method was conducted as previously reported [19].

### Isolation of GMSCs from BRONJ and Healthy Gingival Tissues

GMSCs were isolated from gingiva as previously described [20], GMSCs under 3–5 passages were used. The cell morphology was analyzed with direct microscopic observation and immunofluorescence assay. First, microscopic images of the cells were acquired using an inverted contrast-phase microscope (Nikon, Tokyo, Japan). Then the cells were stained with a fluorescent dye for actin called (Tetramethyl Rhodamine Isothiocynate) TRITC phalloidin (YEASEN, USA). Fluorescence images were obtained using a fluorescence microscope (Olympus, Tokyo, Japan).

### Flow Cytometric

Surface antigens of GMSCs were analyzed by flow cytometry. Briefly, 2 × 10^5^ cells were incubated with mouse anti-human CD45, CD31, CD146, CD90 and CD105 for 30 min at 37^◦^C. Labeled cells were analyzed using a flow cytometer (Beckman, USA).

### Cell Proliferation, Adhesion and Scratch Assay

GMSCs were seeded at a density of 3 × 10^3^ cells/mL into 96-well plate. The cell number was assessed on 1,3,5,7 days using Cell Counting Kit-8 (Beyotime) assay. The optical density was measured at 450 nm using the Spark™ 10 M Multimode Microlpate Reader (TECAN). The experiments of cell adhesion and scratch were done according to previously reported protocol [21]. For cell adhesion, GMSCs were divided into 5.0 × 10^4^ cells/ml and subsequently seeded on to type I collagen coated 6 well plates and incubated for 30 min at 37^。^C. Then the wells were rinsed vigorously three times with phosphate buffered saline (PBS), and the remaining cells were stained using 0.1% crystal violet dye. Data were expressed as adherent cells per field. For cell scratch, GMSCs were plated at 200,000 cells/well in 6-well plates. Once confluent, a scratch wound was performed using a sterile 10 μl pipette tip. The size of the gap was measured microscopically immediately (0 h) and 24 h later.

### Cell Cycle and Apoptosis

GMSCs were seeded at a density of 5 × 10^**3**^ cells/ml into 6-well plate. After cell were detached, cell cycle was analyzed using CycleTESTTM PLUS DNA Reagent Kit (BD, Biosciences). After cells were fixed in 75% ice-cold ethanol, cell apoptosis was analyzed using FITC Annexin V Apoptosis Detection Kit I (BD, Biosciences). Finally, the samples were filtered through 22-µm nylon mesh and evaluated by flow cytometer (Beckman, USA).

### *In Vivo* Wound Healing Assay

#### Lentiviral Vector Transduction

Lentiviral vector PCHMWS-GFP-T2A-Fluc was purchased from Dr. A. Ibrahimi (Katholieke Universiteit Leuven). This vector contained a fused gene encoding for the firefly luciferase (Fluc) and GFP. Briefly, GMSCs were plated at 100,000 cells into 25-cm2 flask. After overnight culture, cells were transduced with 2 ml medium that contained lentiviral vector at 37 °C for 4 h by the multiplicity of infection (MOI) of 20 and then replaced with fresh medium. Three runs of cell transduction were carried out. Four days after the first transduction, the transduced GMSCs reached confluency and were subcultured at a density of 1000 cells/cm^2^ in 150-cm^2^ flasks. After 7 days, when these cultures were near confluency, the GMSCs were cryopreserved at 10^6^/vial (passage 3) at -80 °C. The cells were selected with puromycin (Genomeditech, China) at a low concentration (2 μg /mL) and cultured for 5 days. A GFP (Green Fluorescent Proteins)-positive signal was detected in 95% of the selected cells under an inverted fluorescence microscope (Nikon, Japan).

#### In Mice Skin Wound Healing Model

Luciferase/GFP-labeled GMSCs were implanted in wound healing model as described previously [22–24]. In brief, 5-week-old immunocompromised mice were individually anesthetized using an intraperitoneal injection of ketamine (75 mg/kg) and rinsed with an alcohol swab and sterilely prepped with betadine and draped. A sterile 8 mm diameter full-thickness wound was created on the dorsum of the nude. A donut-shaped splint with a 10 mm inner diameter and 20 mm outer diameter was fashioned from a 0.5 mm-thick silicone sheet (Grace Bio-Laboratories, Bend, OR). An immediate-bonding adhesive (Tegaderm, 3 M) was used to fix the splint to the skin followed by interrupted 5–0 nylon (Ethicon, Inc,Somerville, NJ) sutures to ensure position. Mastisol (Fernadale, MI) was applied to the perimeter of the wound to improve adherence of the occlusive dressing (Tegaderm, 3 M) placed to cover the wounds. The animals were placed in individual cages under a warming lamp and allowed to recover fully from anesthesia. The wound dressings in each group were changed every 3 days according the above methods. 15 mice were randomly divided into three groups: Group A, hydrogel/control GMSCs; Group B, hydrogel/center-BRONJ GMSCs; Group C, hydrogel/peri-BRONJ GMSCs, n = 5. Figure 4 showed the experimental design and schematic representation of wound healing model in nude mice.

#### Bioluminescence Imaging

On days 7 and 14 post transplantation, in vivo cell viability was confirmed by measuring the luciferase activity with a bioluminescence imaging system (IVIS Lumina III, PerkinElmer, USA). Briefly, prior to anesthesia, D-luciferin (potassium salt, Yeasen, China) was injected into the mice at 150 mg/kg. The mice were imaged 20 min after injection. Photon flux was measured and quantified by the system software.

#### Wound Closure Measurements

Every day, nude mice were observed and digital images were taken. Wound area was measured by tracing the wound margin and calculating the pixel area using Image-J 1.52a software (Wayne Rasband, USA). The wound healing rates were calculated as follows: wound closure rate = (A0 − At)/A0 [25]. A0 is the initial wound area, and At is the wound area at 5, 10 and 14 days post-surgery.

#### Histology

After 2 weeks, all mice were sacrificed and the wound tissues were harvested with a rim healthy normal skin tissue. Tissue samples were fixed in 10% formalin. Frozen sectioning and fluorescence microscope detected cell viability as described previously [26]. After imaging by a fluorescence microscope, five random fields were selected to calculate GFP signal areas. Then, the samples were stained with H&E and Masson staining. The lengths of neo-epithelium in H&E staining were calculated according to previously described methods [25]. Masson staining was used to determine the content and maturity of collagen in the wound beds. The fraction of collagen was calculated by detecting the blue area in five random files under the 400 × magnification fields of each group using Image-J 1.52 software. IH staining was the same as the previous experiment.

### cDNA Microarray

Gene expression profiling was performed using the Affymetrix GeneChip (Affymetrix, Santa Clara, CA, US). The details of RNA sample extraction and quality control were in Supplement 2. Raw data were normalized by RMA algorithm, Affymetrix packages in R. Differentially expressed genes were selected at ≥ twofold and p < 0.005. Gene Ontology (GO) analysis and Kyoto Encyclopedia of Genes and Genomes (KEGG) analysis were performed using the clusterProfler R/bioconductor package version 3.16.0. Only pathways with ≥ 2 genes were included in the analysis. P-values of hypergeometric tests were adjusted for multiple testing via the Benjamini–Hochberg method. For all pathways with adjusted P‑value ≤ 0.05.

### RT-PCR

Total mRNA was isolated using TRIzol reagent (Invitrogen Life Technologies), cDNA was prepared using GoScript Reverse Transcription System (Promega), and an ABI Prism 7500 (Bioscience) was used to perform RT-PCR. The relative mRNA expression levels was determined by normalizing to the β-Actin threshold cycle and calculated using the ^△△^Ct method. Primers are shown in Supplement 3.

### Western Blot

Proteins were extracted from GMSCs, and Western blot assays were performed as previously described [27]. Primary antibodies against β-actin, TGF-β1(Abcam; 1:1000), COLIA1(Abcam; 1:1000) and p-Smad3 (Cell Signaling Technology; 1:1000) were used.

### Statistical Analysis

All statistical analysis was performed using GraphPad Prism 7 (GraphPad Software, USA). The outcome measurements are expressed as the mean ± standard deviation (SD). Differences between two groups were analyzed by t-test. P ≤ 0.05 was considered as the statistically significant difference for all comparisons. All experiments were conducted in triplicate.

## Results

### Histological Assessment of Healthy and BRONJ Patients’ Gingival Tissues

**Fig. 1:**
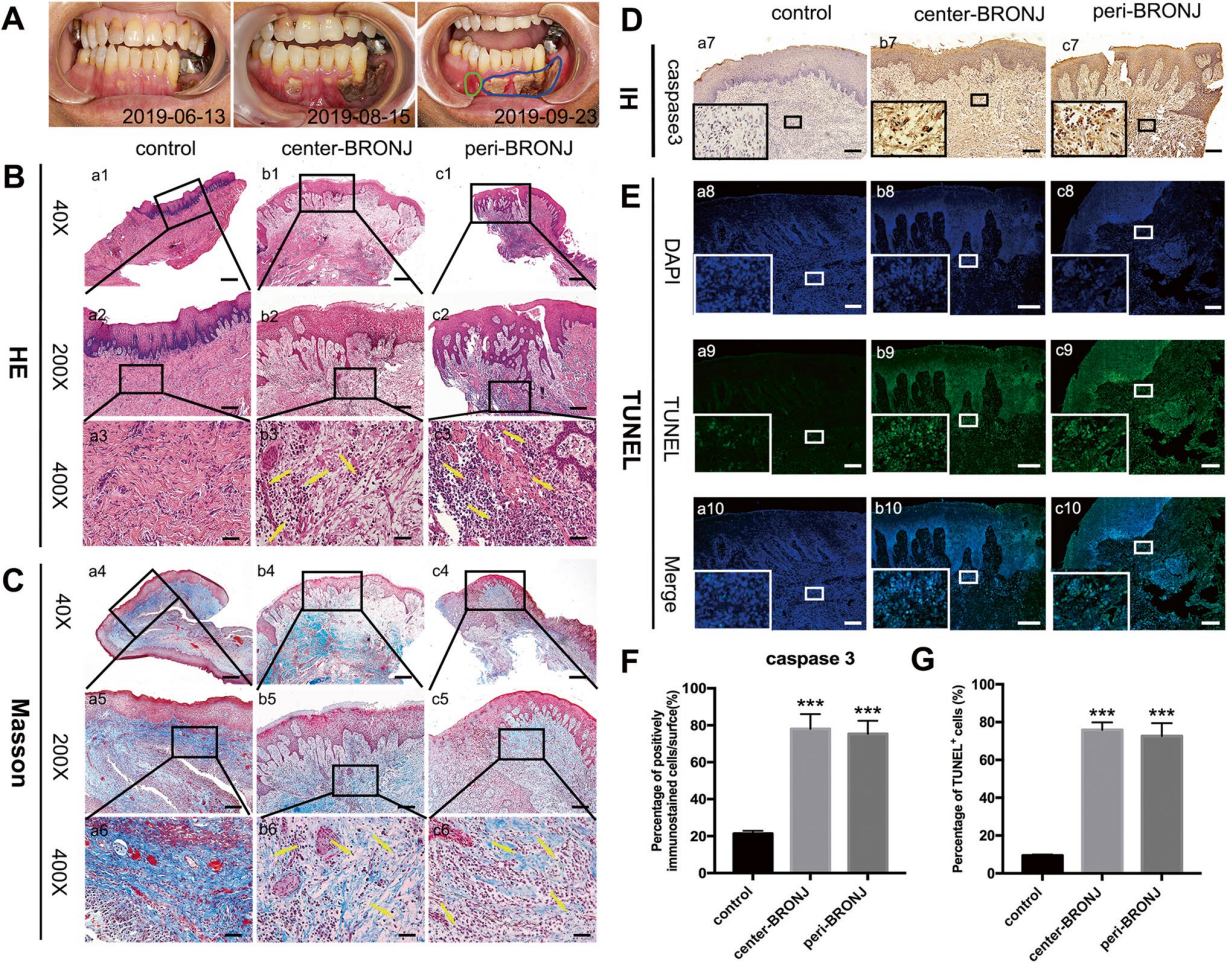
Histological analysis of gingival tissues from BRONJ patients and healthy people. **A** The progress of a BRONJ patient within 3 months, showing the progressive enlargement of the gingiva defect. The blue circle represents the central area of BRONJ, and the green circle denotes the peripheral area from where the gingival samples were acquired. **B** Representative images of HE staining of gingival tissues in each group. **C** Masson staining of gingival simples. **D** Immunohistochemistry showing caspase 3 expression in different groups and **F** quantified expression. **E** TUNEL staining of gingival samples and (**G**) statistical analysis in each group. (a1–c1, a4–c4, scale bar = 500 μm; a2–c2, a5–c5, a7–c7, scale bar = 100 μm; a3–c3, a6–c6, a8-10–c8-10, scale bar = 50 μm. *** p < 0.001)

Figure 1A showed the progressive enlargement of the gingiva defects in a BRONJ patient within 3 months. The blue circle represents the gingival tissue in the central area of BRONJ lesion, and the green circle represents the peripheral area (standard debridement boundary [17]) from where the following samples were required. Gingival samples were assessed following HE staining. In contrast with healthy gingiva, the central and peripheral area of BRONJ gingival tissues were infiltrated with abundant lymphocyte and plasma cells (yellow arrow), and appeared as irregular, serrated spikes (Fig. 1B). To investigate the collagen deposition in each group, Masson staining indicated that BRONJ gingiva also displayed the disorganized, cracked, loose lamina propria and a major reduction of collagen fibers (yellow arrow) compared with healthy gingiva (Fig. 1C). Moreover, IH staining (Fig. 1D) and quantified expression (Fig. 1F) showed remarkably increased expression of caspase 3 (the apoptotic executioner) in BRONJ gingival tissues (Fig. 1D). Apoptosis was also evaluated as the number of TUNEL + cells in different groups (Fig. 1E), similar levels of TUNEL + cells existed between the center and peripheral area of BRONJ lesions, but the apoptotic cells in BRONJ lesions were significantly higher than in healthy gingival tissues (Fig. 1G). Taken together, these results suggest that there is an association between the microenvironment of BRONJ patients’ gingiva and the impaired gingival wound healing.

### Isolation of GMSCs from BRONJ Patients’ Gingiva and Healthy Gingiva

**Fig. 2:**
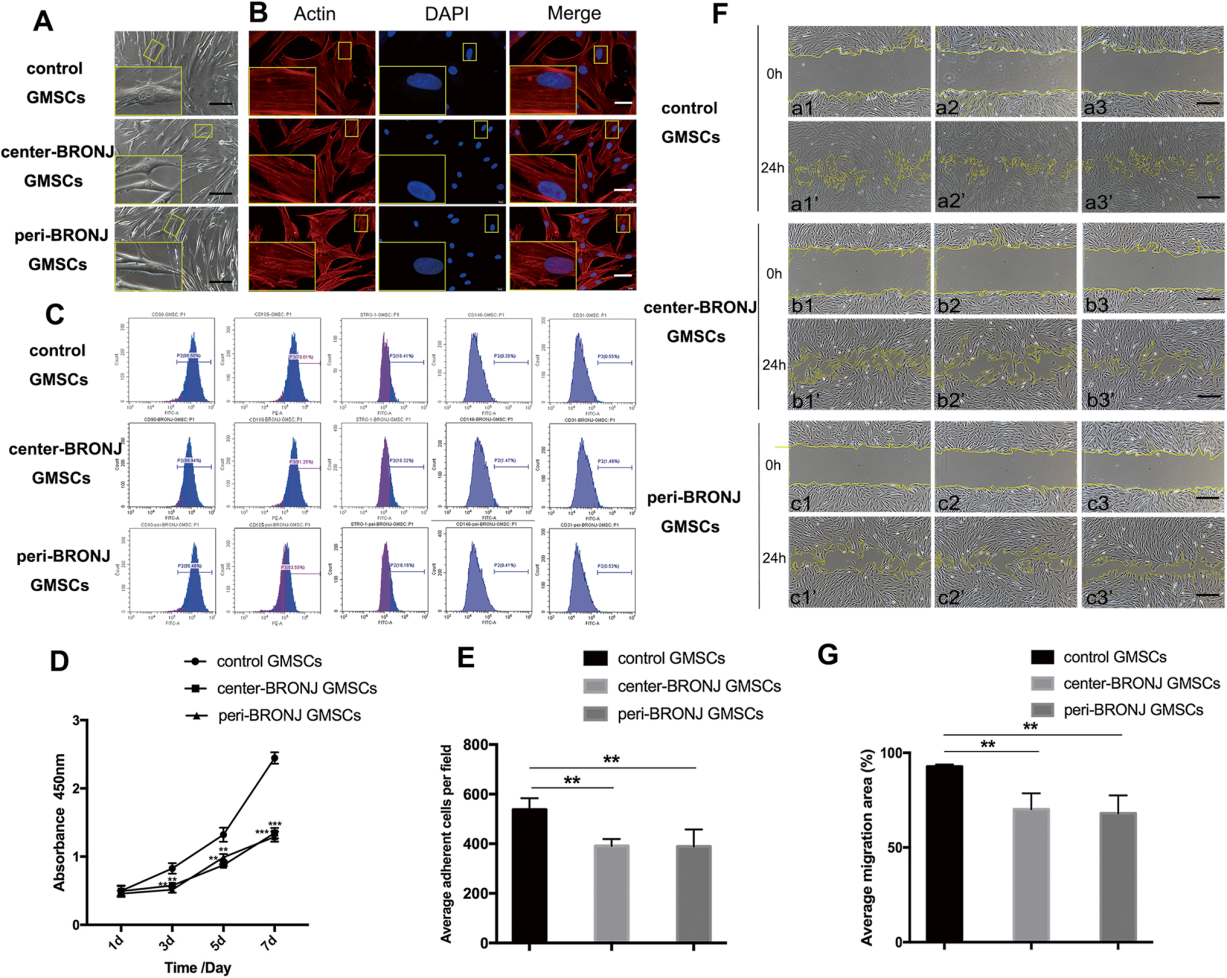
Isolation and identification of gingival marrow stem cells (GMSCs) from BRONJ patients’ gingiva. **A** GMSCs derived from gingival tissues in the central area of the BRONJ region (center-BRONJ GMSCs), the peripheral area (peri-BRONJ GMSCs) and control patients (control GMSCs) were all fibroblast-like cells, but BRONJ GMSCs became slender and wrinkled, resembling ice crystals (scale bar = 500 μm). **B** Immunofluorescence assay for actin fiber evaluation (scale bar = 50 μm). **C** GMSCs identification by flow cytometry analysis. **D** Comparison of proliferative abilities of GMSCs were analyzed at 1–7 days. **E** Quantitative analysis of average adhesion cell count per field. **F** Scratch wounds were directly microscopic observed at 0 and 24 h. a1, a2, and a3 represent 3 different control patients. b1 and c1, b2 and c2, and b3 and c3 represent 3 BRONJ patients. The a, b, c represent the 0 h images and a’, b’, c’ represent the 24 h images (scale bar = 500 μm). **G** Quantitative evaluation of average migration area (**p < 0.01, ***p < 0.001)

The stromal cells were derived from gingival tissues in the central area of BRONJ region (center-BRONJ GMSCs), peripheral area (peri-BRONJ GMSCs) and health gingiva (control GMSCs). These cells were all fibroblast-like cells, but the central and peripheral BRONJ GMSCs from all 5 BRONJ patients all became slender and more wrinkled, resembling ice crystals (Fig. 2A). Consistent with the microscopic observation, fluorescence images (Fig. 2B) of actin fibers in control GMSCs showed a dense and aligned network-like structure throughout the whole of the cell body, while the central and peripheral BRONJ GMSCs became atrophic and spindle-shaped morphology with long hair-like actin fiber. But no significant differences were found between the center-BRONJ GMSCs and peri-BRONJ GMSCs. The flow cytometry results (Fig. 2C) showed that these cells were all positive for mesenchymal stem cell (MSC)–related markers STRO-1, CD90, and CD105.

### BRONJ GMSCs Exhibited Poor Proliferation, Adhesion and Migration Ability than Control GMSCs

The cell growth curves are illustrated in Fig. 2D. Compared with control GMSCs, the central and peripheral BRONJ GMSCs all showed a lower proliferation, but no significant differences were found between center-BRONJ GMSCs and peri-BRONJ GMSCs. Cell adherent analysis (Fig. 2E) demonstrated that the number of adherent cells was fewer in the central and peripheral BRONJ GMSCs than control GMSCs within 4 h, while no significant difference between center-BRONJ GMSCs and peri-BRONJ GMSCs was evident. Moreover, direct microscopic observation in cell migration (Fig. 2F) performed that the peri-BRONJ GMSCs exhibited the slowest cell migration rate and center-BRONJ GMSCs showed a lower migration rate than control GMSCs after 24 h (the average migration area in center-BRONJ GMSCs: 70.23% ± 8.38% vs. peri-BRONJ GMSCs: 68.05% ± 9.51% vs. control GMSCs: 92.83% ± 1.04%, respectively) (Fig. 2G). These data suggested that the abilities of proliferation, adhesion, and migration of the central and peripheral BRONJ GMSCs were all remarkably decreased compared with controls.

### BRONJ GMSCs were Arrested Cell Cycle in G0/G1-Phase and Underwent Early Apoptosis Compared with Control GMSCs

**Fig. 3:**
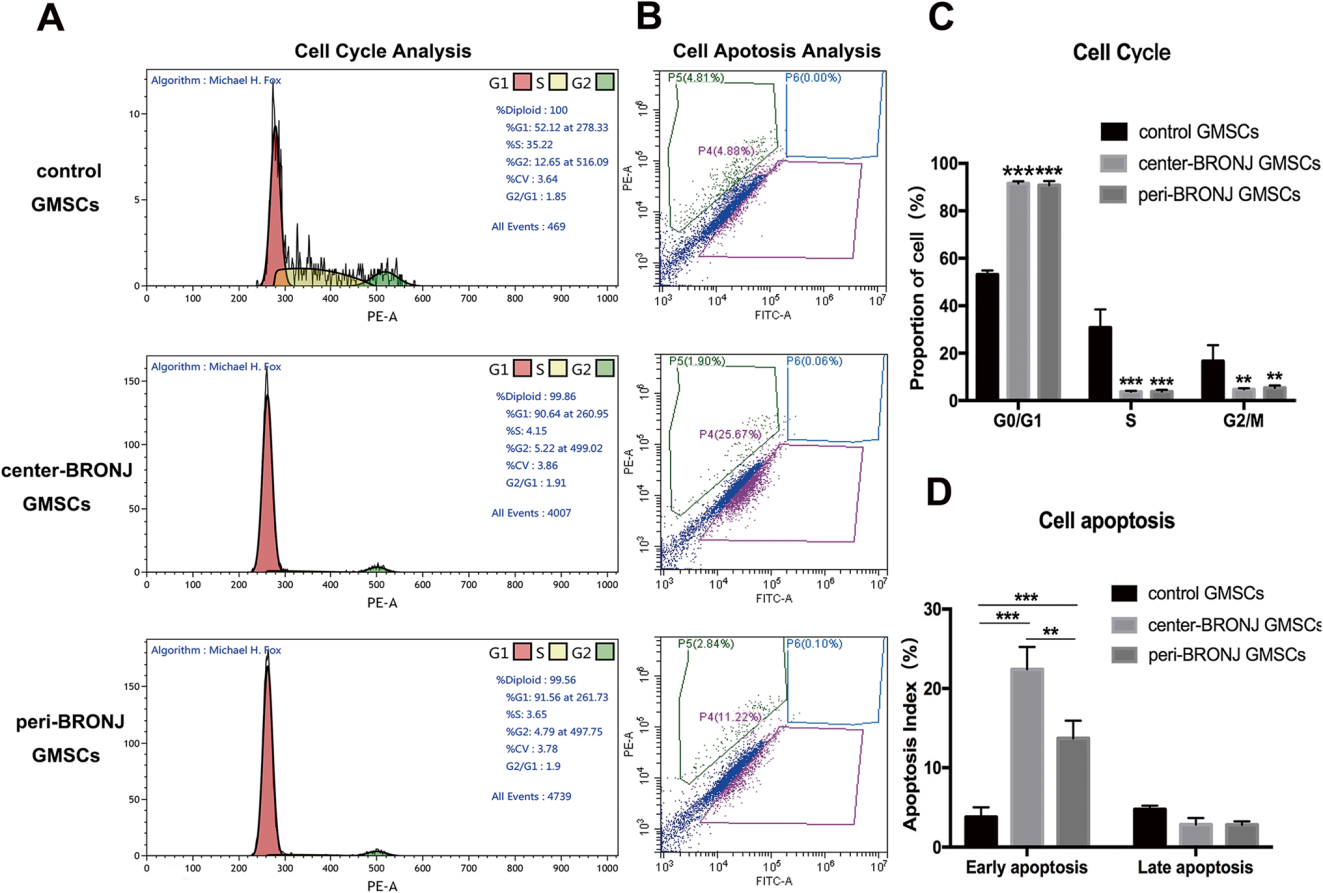
BRONJ GMSCs were arrested cell cycle in G0/G1-phase and underwent early apoptosis. **A** Cell cycle and **B** apoptosis were determined by flow cytometric analysis in center-BRONJ GMSCs, peri-BRONJ GMSCs and control GMSCs. **C** Quantitative analysis of the percentage of GMSCs in each cycle phases and **D** the rate of early apoptotic cells in different groups (**p < 0.01, ***p < 0.001)

We further investigate cell cycle and apoptosis in all groups according to flow cytometry. Results of the percentage of cells in each cell cycle phase are depicted in Fig. 3A, the percentage of cells in G0/G1 phase was significantly increased in the central and peripheral BRONJ GMSCs compared with controls (Fig. 3C). Furthermore, Fig. 3B presents the percentage of cell apoptosis in all groups. From cell apoptosis analysis in Fig. 3D, it is apparent that center-BRONJ GMSCs showed the highest percentage of early apoptotic cells and peri-BRONJ GMSCs showed higher rate comparison with control GMSCs (the early apoptotic cells rate in center-BRONJ GMSCs: 22.41% ± 2.82% vs. peri-BRONJ GMSCs: 13.73% ± 2.22% vs. control GMSCs: 3.77% ± 1.23%, respectively). Taken together, these results indicated that the central and peripheral BRONJ GMSCs were all arrested cell cycle in G0/G1-phase and underwent early apoptosis compared with controls.

### BRONJ GMSCs Showed Significantly Lower Cell Viability *In Vivo*

**Fig. 4:**
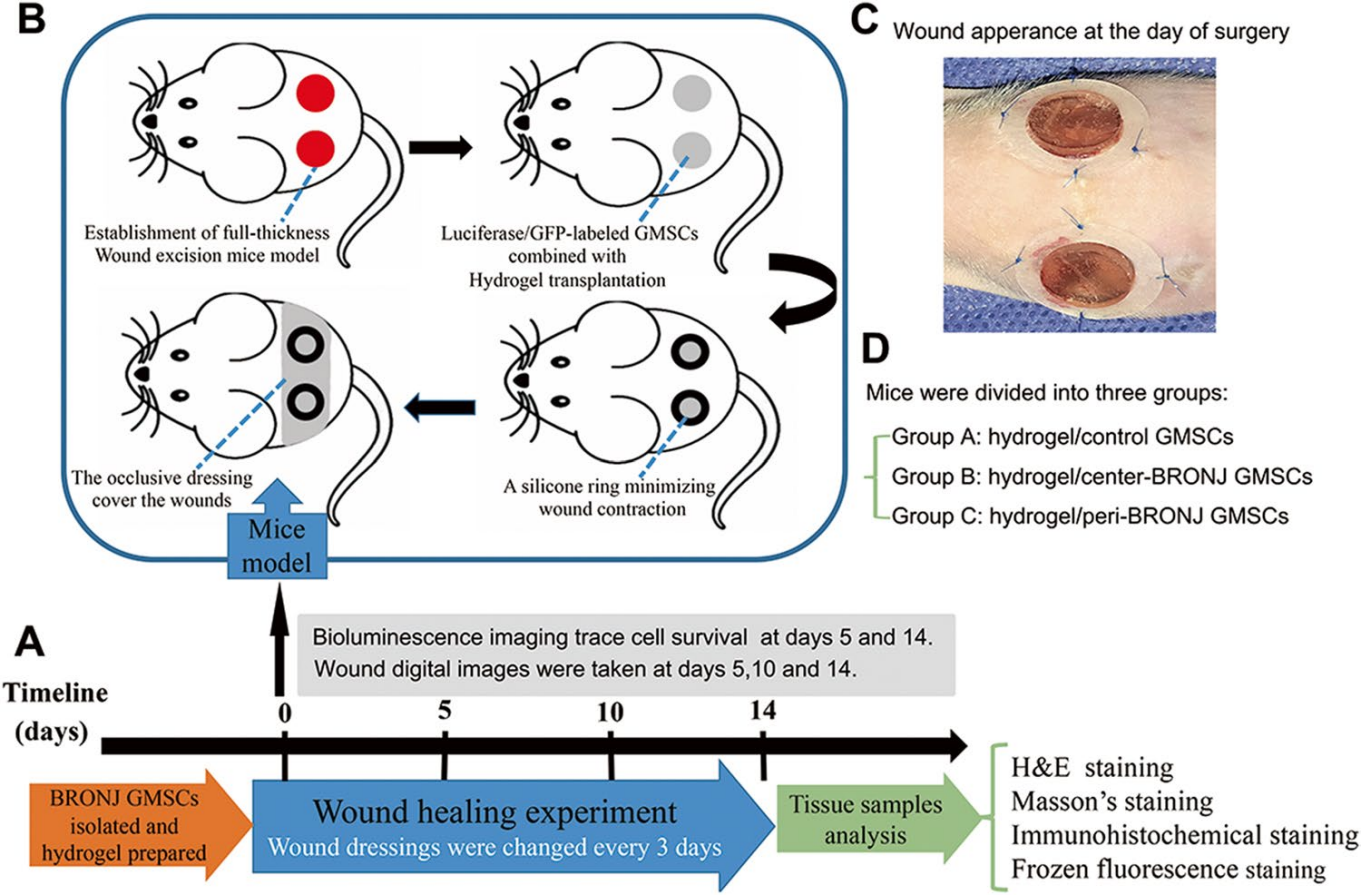
Animal experimental design for skin wound healing and the in vivo studies. **A** Timeline describing the in vitro cell isolated, hydrogel prepared and the mice wound healing experiment. **B** Building full-thickness excisional wound healing model and Luciferase/GFP-labeled GMSCs combined with hydrogel transplant into the wound site. **C** Wound appearance in nude mice at the day of surgery. **D** Mice were divided into three groups: Group A, hydrogel/control GMSCs; Group B, hydrogel/center-BRONJ GMSCs; Group C, hydrogel/peri-BRONJ GMSCs, n = 5

**Fig. 5:**
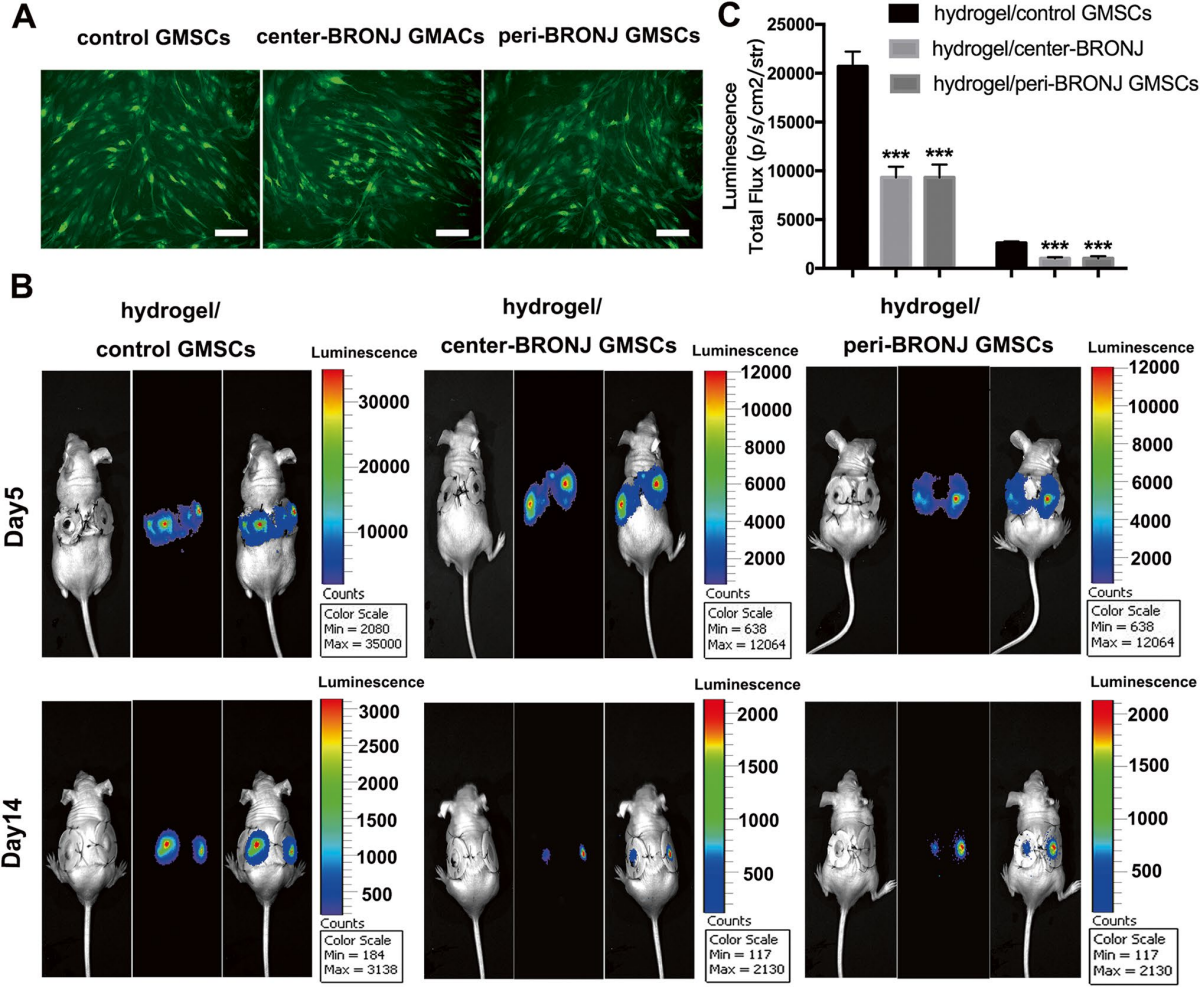
BRONJ GMSCs showed significantly lower cell viability in vivo. **A** Green fluorescent protein expression in GMSCs was observed after transduced by lenti-Luc/GFP 72 h (scale bar = 50 μm). **B** The Luciferase/GFP-transduced GMSCs combined with hydrogel were transplanted in skin wound bed in mice. Cell viability and proliferation in vivo were measured by bioluminescence imaging on days 5 and 14. **C** Statistical analysis of the photon flux representing the viability of transplanted cells in mice (n = 5, ***p < 0.001)

Figure 4 showed the experimental design and schematic representation of wound healing model in nude mice. The GFP fluorescence reached peak levels 72 h after lentiviral transduction in different groups (Fig. 5A). Hydrogel [25] laden with luciferase/GFP-labeled GMSCs were transplanted in skin healing model in nude mice to trace cell survival. Bioluminescence imaging (Fig. 5B) showed significant differences in different groups on days 5 and 14, performing significantly lower cell survival rate in BRONJ GMSCs groups than that in hydrogel/ control GMSCs group, while there was no statistical difference between the hydrogel/center-BRONJ GMSCs group and hydrogel/peri-BRONJ GMSCs group (Fig. 5C).

### BRONJ GMSCs Transplantation Exhibited Poor Wound Healing Effect than that of Control GMSCs in Mice Model

**Fig. 6:**
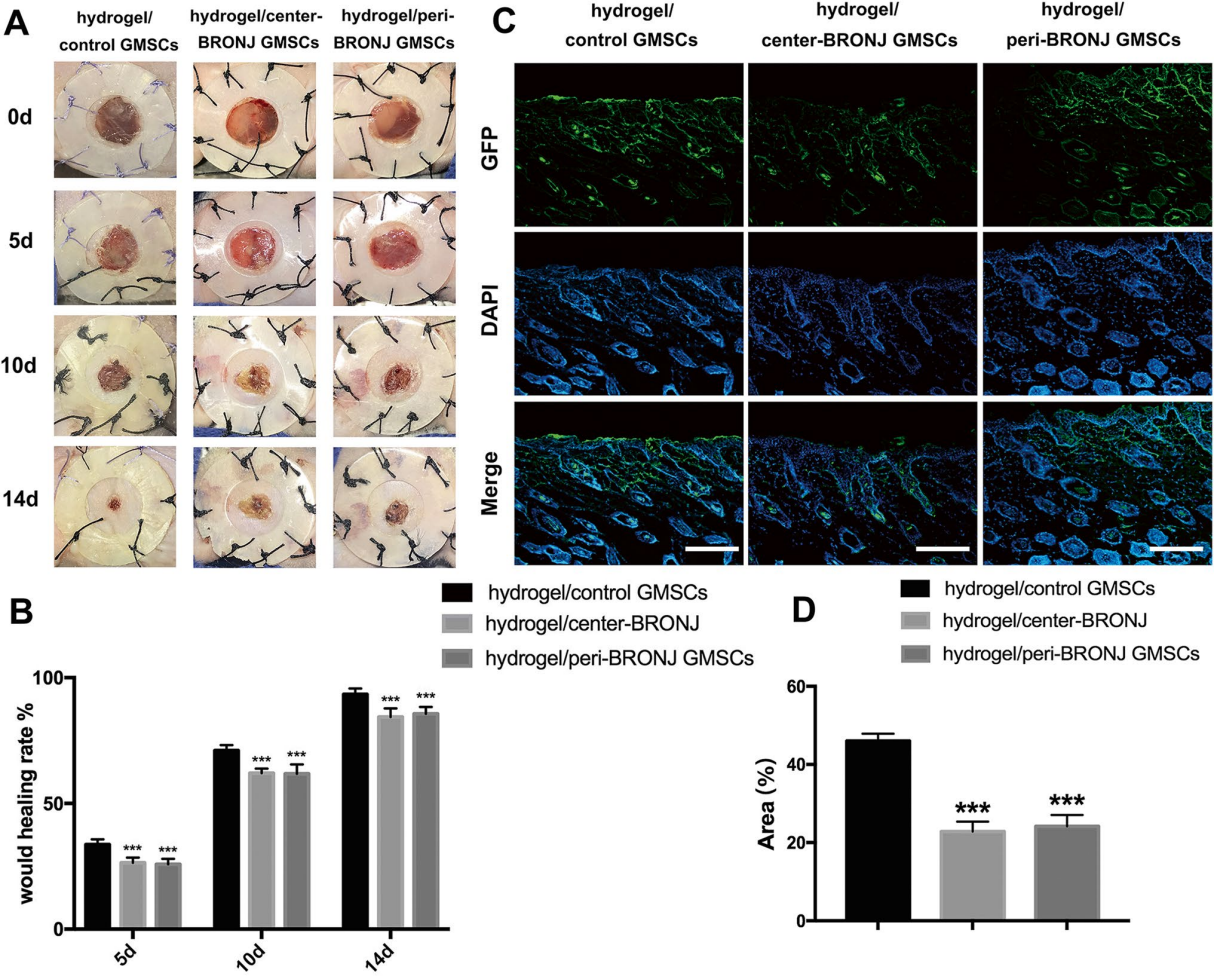
Macroscopic appearances and quantitative analysis of cutaneous wounds in the different groups. **A** Representative images of full-thickness skin defects in nude mice at hydrogel/control GMSCs group, hydrogel/center-BRONJ GMSCs and hydrogel/peri-BRONJ GMSCs group. **B** Quantitative analysis of the wound healing rates in each group at 5, 10 and 14 days post-surgery (n = 5, ***p < 0.001). **C** Frozen sectioning and IF staining of cutaneous wound samples extracted on day 14 (scale bar = 100 μm). **D** Statistical analysis of GFP signal areas was based on 5 randomly selected high-power fields representing the viability of transplanted cells (n = 5, ***p < 0.001)

**Fig. 7:**
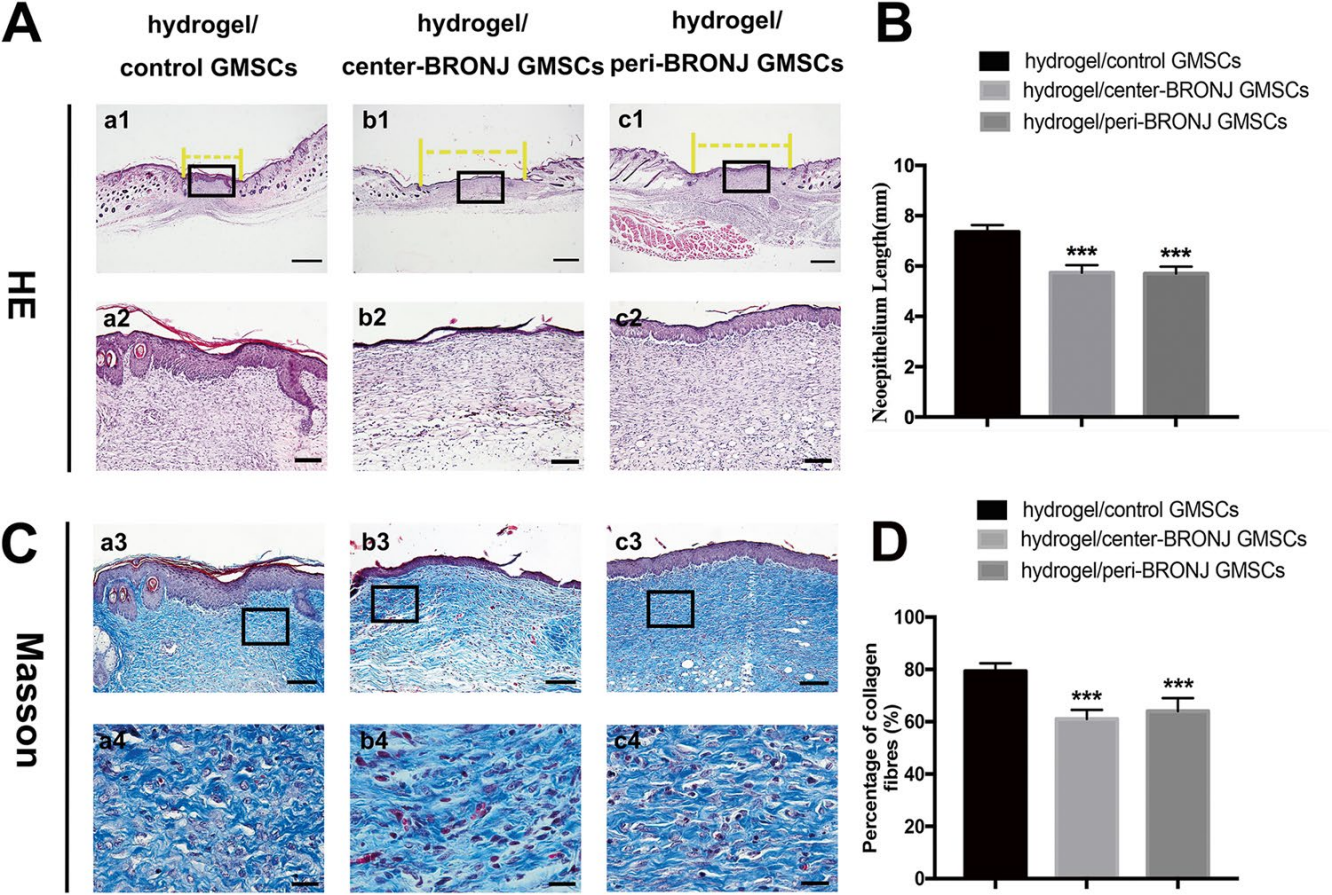
Histologic analysis of the wound sections. **A** Representative images of H&E staining of the wound sections in each group. The yellow dotted line indicates the length without re-epithelialization in the wound. **B** Quantitative analysis of the neo epithelialization in the four groups at 14 days post-surgery. **C** Representative images of Masson staining in different groups. **D** Quantitative analysis of the percentage of collagen in each group (a1–c1, scale bar = 500 μm; a2–c2, a3–c3, scale bar = 100 μm; a4–c4, scale bar = 20 μm. n = 5, ***p < 0.001)

Given the essential role of GMSCs in gingival wound healing, we evaluated the wound healing effects of BRONJ GMSCs using an excisional skin healing model in nude mice. Figure 6A shows optical images of all groups at 0, 5, 10 and 14 days post-surgery. Obviously, the wound size of hydrogel/ control GMSCs group was the smallest compared to the other two groups, and the wounds had almost closed by 14 days. Remarkably, wound closure was slower both in the center and peri-BRONJ GMSCs groups compared with control group, while no significant differences were found between the center-BRONJ GMSCs group and peri-BRONJ GMSCs group. Quantitation of the cutaneous wound size confirmed the above results (Fig. 6B). Furthermore, when investigating the samples by frozen sectioning and immunofluorescent (IF) staining in excisional wound beds (Fig. 6C), we also found lower cell viability both in the center and peri-BRONJ GMSCs groups than that of control group (Fig. 6D). Moreover, histological analysis showed the neo-epithelium in the cutaneous wound defects in all groups (Fig. 7A), and the yellow line indicated the length without re-epithelialization in the wound. As can be seen from the Fig. 7B, the total neo-epithelium length in hydrogel/ control GMSCs group was significantly longer than BRONJ GMSCs groups, but there was no statistical difference between the center-BRONJ GMSCs group and peri-BRONJ GMSCs group. Masson’s staining (Fig. 7C) and quantitative analysis were further applied to evaluate the collagen deposition and maturation. In detail, extensive deposition of collagen fibers was obviously observed in the wound bed of the hydrogel/ control GMSCs group compared with the BRONJ GMSCs groups. Consistently, quantitative analysis (Fig. 7D) also revealed that the content of the collagen in control group was significantly higher than the other two groups, however, there were not statistically significant difference between the two groups. These results suggest that BRONJ GMSCs transplantation exhibited poor wound healing effect than that of control GMSCs in mice full-thickness excisional skin healing model in vivo.

### Microarray Gene Profiling Identifies the Negative Regulation of Wound Healing and the Suppressed TGF-β Signaling Pathways

**Fig. 8:**
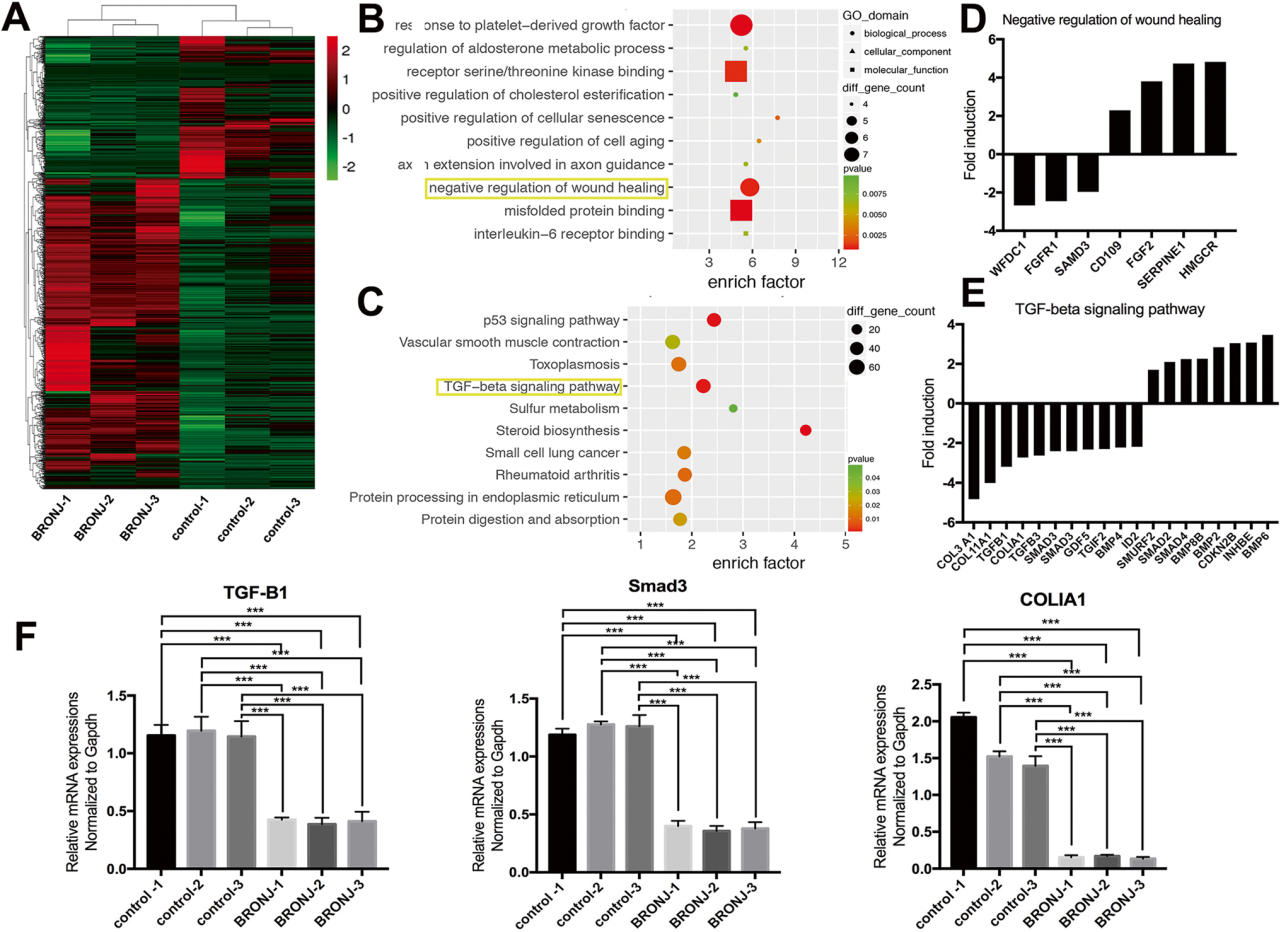
Microarray gene profiling identifies the activation of the negative regulation of wound healing and the suppressed TGF-β signaling pathways. **A** A graphic representation of the microarray profiling that are differentially expressed more than threefold were represented (n = 3). **B** GO enrichment analysis identified “negative regulation of wound healing” as one of the most significantly associated biological process in BRONJ lesions. **C** Kyoto Encyclopedia of Genes and Genomes (KEGG) analysis showed “TGF-β signaling pathways” as the significantly enriched functional pathway associated with the retarded gingival wound healing. **D** Lists of genes involved in negative regulation of wound healing. **E** Lists of genes involved in TGF-β signaling pathways. **F** BRONJ gingival tissues and healthy gingiva were harvested and subjected to RT-PCR analysis. The results are from three different BRONJ patients and three control people (n = 3, ***p < 0.001)

To explore the underlying mechanisms of the retarded gingival wound healing in BRONJ patients, we performed an Affymetrix Gene Expression Array analysis using tissues obtained from three BRONJ patients and three healthy people respectively, and explored differentially regulated genes (biological replicate, n = 3). BRONJ gingival tissues were subjected to microarray profiling, and differentially expressed genes that were either upregulated or downregulated more than twofold were obtained (Fig. 8A). GO enrichment (Fig. 8B) in analysis [28] identified “negative regulation of wound healing” as one of the most significantly associated biological process in the development of BRONJ, in which fibroblast growth factor receptor 1 (FGFR1) and Smad3 were all downregulated (Fig. 8D). KEGG analysis [29] showed that “TGF-β signaling pathways” were the significantly enriched functional pathways associated with the retarded gingival wound healing in BRONJ patients (Fig. 8C). Involved in this signaling pathways, expression of COLIA1, COL3A1, COL11A1 and TGF-β1, TGF-β3, Smad3 were highly downregulated (Fig. 8E).

### TGF-β1 Signaling Pathway was Suppressed not only in BRONJ Patients’ Gingiva but in BRONJ GMSCs Transplantation Animal Model

**Fig. 9:**
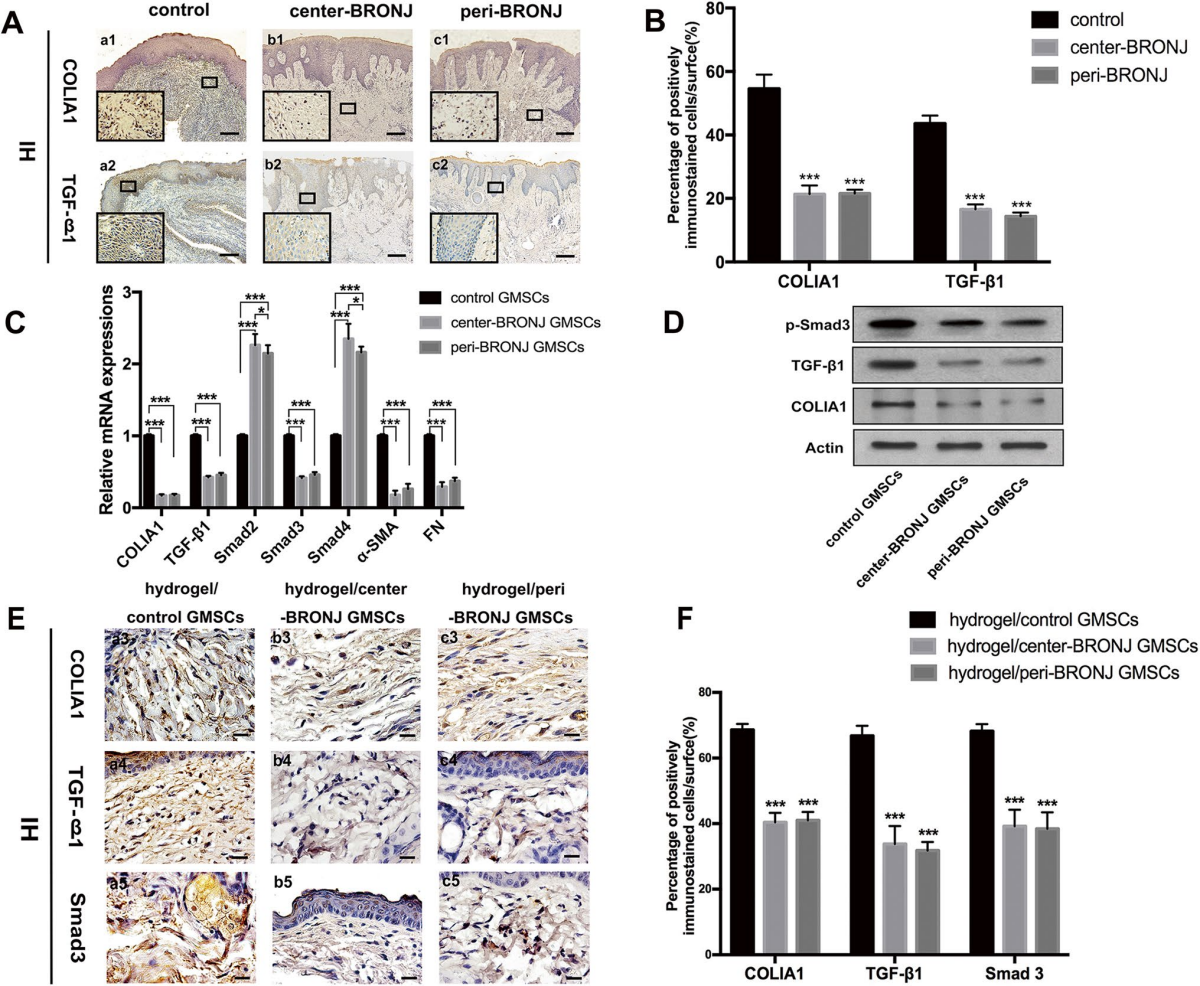
Downregulation of TGF-β1 signaling both in BRONJ patients’ gingiva and BRONJ GMSCs transplantation animal model. **A** Immunohistochemical staining of COLIA1 and TGF-β1 in different groups and (**B**) quantitative analysis of their expressions in patients’ gingival tissues (a1–c1, a2–c2, scale bar = 100 μm. The lower left corner in each image with a magnification of 50 × . n = 5, ***p < 0.001). **C** The mRNA levels of GMSCs in each groups were determined by real-time PCR (n = 5, *p < 0.05, ***p < 0.001). **D** Western blot showed the protein levels of TGF-β1, p-Smad3 and COLIA1 in different GMSCs. **E** Immunohistochemistry to detect COLIA1, TGF-β1, Smad3 expressions in each group and (**F**) quantitative analysis in the cutaneous wound beds in mice (a3–c3, a4–c4, a5–c5, scale bar = 20 μm, n = 5, ***p < 0.001)

To verify the microarray results, firstly, we validated the suppressed expression of TGF-β signaling pathway in BRONJ patients’ gingiva. The mRNA levels of BRONJ gingiva were determined by real-time PCR, we found that the expression of TGF-β1, Smad3, COLIA1 were remarkably downregulated in BRONJ gingiva (Fig. 8F). The same pattern was further confirmed by IH staining, which showed a lower expression of TGF-β1, and COLIA1 in the central and peripheral area of BRONJ sites than that in healthy gingiva (Fig. 9A). In addition, the mRNA levels of the center-BRONJ GMSCs and peri-BRONJ GMSCs were also determined by real-time PCR. Consistently, TGF-β1, Smad3 and COLIA1 were also the most significantly downregulated both in the central and peripheral BRONJ GMSCs compared with controls, however, no significant difference between the two groups was evident (Fig. 9C). Western blot analysis further confirmed that TGF-β1, p-Smad3 and COLIA1 were expressed at a lower level both in the central and peripheral BRONJ GMSCs than that in the controls (Fig. 9D). Thus, we speculated that BRONJ GMSCs were associated with impaired gingival healing may partly via suppressing TGF-β1 signal pathway.

According to the results in mice skin healing model, the wounds had mostly closed in the control group but not in the BRONJ GMSCs groups at 14 days post-surgery, which suggest that BRONJ GMSCs decelerate wound healing within 14 days post-surgery compared with control GMSCs. To further explore the exact mechanism by which BRONJ GMSCs delay primary wound healing, IH staining was performed to detect the fibrosis in the wound beds (Fig. 9E), performing that COLIA1, TGF-β1 and Smad3 were the most significantly downregulated in the center-BRONJ GMSCs and peri-BRONJ GMSCs groups compared with the controls, however, there was no significant difference between the two groups (Fig. 9F). Consistent with our observation in BRONJ patients’ gingival samples, these results further corroborated the suppressed expressions of TGF-β1 pathway in BRONJ GMSCs transplantation animal model. According to the findings, we can infer that BRONJ GMSCs transplantation in mice exhibited poor wound healing effects than that of control GMSCs may also partly by downregulating TGF-β1 signaling pathway. Taking into consideration of the above results, we re-affirmed that TGF-β1 signaling pathway was suppressed not only in BRONJ patients’ gingiva but in BRONJ GMSCs transplantation animal model.

## Discussion

In this study, we successfully isolated GMSCs from the central area of BRONJ patients’ gingiva and the peripheral area at the recommended debridement boundary. We demonstrated for the first time that the center-BRONJ GMSCs and peri-BRONJ GMSCs showed decreased proliferation, adhesion, migration capacities and underwent early apoptosis in vitro compared with control GMSCs. In addition, the central and peripheral BRONJ GMSCs transplantation in a mice excisional skin model consistently exhibited poor wound healing effects than that of control group. Mechanistically, TGF-β1 signaling pathway was suppressed not only in BRONJ patients’ gingiva but in BRONJ GMSCs transplantation animal model. Our findings highlight the dysfunction of BRONJ GMSCs and their roles in impaired gingival wound healing, further providing new insights into the prevention of BRONJ.

The key clinical feature of BRONJ is the retarded gingival healing with necrosis exposure [30], however, it is still unclear why such the lesion should present with loss of oral soft tissue as the primary clinical feature. As mentioned in the literature, oral mucosa as a unique soft tissue is superior in terms of preventing wound infection and promoting the underlying bone remodeling [31]. In the past several years there have been numerous reports demonstrating the direct toxic effect of BPs on oral soft tissue [9, 32, 33]. Indeed, in present study, we clinically observed the impaired gingival healing and even the progressive enlargement of gingiva defects in BRONJ patients, histologically manifested by disordered lamina propria and notably depressed expressions of collagen. Prior studies have noted that gingival wound healing properties—rapid re-epithelialization and fetal-like scarless healing—are driven primarily by GMSCs [12, 34]. Moreover, BPs toxicity directly to healthy GMSCs in vitro has been well documented, however, to our knowledge, there is overall lack of direct evidence demonstrating the characterization of GMSCs derived from BRONJ patients’ gingiva as well as the roles in impaired gingival healing. In this study, we first isolated GMSCs from the center area of BRONJ gingiva and peripheral area, and showed that they were positive for MSC related markers STRO-1, CD90, and CD105. They were all fibroblast-like cells, but the center-BRONJ GMSCs and peri-BRONJ GMSCs became slender and more wrinkled, resembling ice crystals. In addition, the cytoskeletal stress fibers in the central and peripheral BRONJ GMSCs also became atrophic and spindle-shaped morphology with long hair-like actin fiber. As we all know, the cytoskeletal stress fiber structure plays essential roles in cellular functions such as shape maintenance and active motility [35]. On the basis of this observation, we speculated that the cellular functions in the central and peripheral BRONJ GMSCs must be impaired.

As expected, we demonstrated that the capabilities of proliferation, adhesion and migration in the central and peripheral BRONJ GMSCs all remarkably decreased compared with controls. The findings were consistent with that of Y. Zhang et al. who reported the proliferative rate of BRONJ BMSCs from both the central and peripheral regions dramatically decreased [36]. Our previous research using BRONJ patients’ periodontal tissue has demonstrated the increased expression of caspase 3 in BRONJ sites, especially in periodontal ligament [3]. Caspase 3, as the apoptotic executioner, is a key enzyme in the apoptotic cascade as it cleaves and activates procaspases 2, 6, 7, and 9 as well as mediating DNA condensation, DNA fragmentation, and cell blebbing [37]. Similarly, in this study, we also observed the remarkably increased expression of caspase 3 in BRONJ gingiva. Apoptotic cells in BRONJ gingival tissues were also detected by TUNEL assay, we found that the proportion of apoptotic cells (TUNEL^+^ cells) in the center area of BRONJ lesions and the peripheral area were all higher than in health gingiva, while no statistical difference was found between the center area of BRONJ lesions and the peripheral area. Furthermore, flow cytometry also demonstrated that the central and peripheral BRONJ GMSCs were arrested cell cycle in G0/G1-phase and underwent early apoptosis compared with controls. Surprisingly, center-BRONJ GMSCs showed a higher early apoptotic rate than peri-BRONJ GMSCs. The possible reason may be that the number of cells (DAPI) in peripheral-BRONJ appears less than center-BRONJ. So, the number of TUNEL^+^ cells in the center group was higher than that in the surrounding group, but the proportion of TUNEL^+^ cells in the two groups was similar. Taken together, these results suggest that the central and peripheral BRONJ GMSCs all exhibit poor proliferation, adhesion, migration ability and undergo early apoptosis, which may offer early evidence for retarded gingival wound healing in BRONJ patients. What is surprising is that no differences are found in the center-BRONJ GMSCs and peri-BRONJ GMSCs. A possible explanation for this might be that under the microenvironment of oral mucosa at the site of BRONJ patients’ lesions, even at the recommended boundary of debridement, the functions of almost all GMSCs are equally impaired, which might help further elaborate why debridement of BRONJ lesions still lead to retarded gingival wound healing or even further aggressive enlargement of soft tissue defects.

We further assessed cell vitality and wound healing capacity of BRONJ GMSCs in vivo by transplanting the center-BRONJ GMSCs and peri-BRONJ GMSCs with hydrogel into full-thickness wound sites of nude mice, which is the most commonly used animal model for excisional wound healing [22]. Using this animal model, the effect of BRONJ GMSCs on wound healing was shown, displaying the significantly lower cell viability in vivo both in the central and peripheral BRONJ GMSCs groups, when compared to the control group. Consistent with our wound healing capacity in vitro, BRONJ GMSCs transplantation in mice also had poor effects on wound healing compared with control GMSCs. To the best of our knowledge, no published studies testing the cell vitality and wound healing capacity of BRONJ GMSCs in a more appropriate animal model have been reported, so our work is novel, and provide infallible testimony about the impaired regenerative ability of BRONJ GMSCs in vivo.

Surprisingly, our study did not find a significant difference between the center-BRONJ GMSCs and the peri-BRONJ GMSCs both in vitro and in vivo. As for the possible reasons, based on our experimental and clinical results, we infer that the altered characteristics of GMSCs from BRONJ patients are primarily due to long-term treatment with BPs from blood support as well as the underlying bone and less dependent on their proximity to the wound site. First, there is still no method to quantify BPs from human skeleton, in particular from BRONJ sequestrations [38]. In our previous clinical retrospective study, we found that the concentration of bisphosphonates in the bone may be positively correlated with radiodensity [39]. In our study, there were no significant difference of the radiodensity between the central area of BRONJ and the peripheral area. So, we speculate that the concentrations of bisphosphonates in the blue circle (central area) and in the red circle (peripheral area) were no significant difference. Second, it is well known that the concentration of BPs in the circulation is uniform at all sites of the blood vessels in the oral. Therefore, we once again reiterated that the center-BRONJ GMSCs and peri-BRONJ GMSCs are equally impaired in BRONJ patients.

To better explore the exact mechanism of the impaired gingival wound healing in BRONJ patients, we performed an Affymetrix Gene Expression Array analysis using BRONJ patients’ gingival tissues. GO enrichment analysis showed that “negative regulation of wound healing” as one of the most significantly associated biological process. KEGG analysis revealed that “TGF-β signaling pathways” as the significantly enriched functional pathway associated with the retarded gingival wound healing. Indeed, numerous studies have reported that TGF-β signaling pathway plays a crucial role in wound healing by controlling collagen synthesis [40–42]. In present study, our IH staining verified that TGF-β1 signaling pathway was remarkably suppressed not only in BRONJ patients’ gingiva but in BRONJ GMSCs transplantation animal model. TGF-β1 is a pleiotropic cytokine with a crucial role in mediating the differentiation and proliferation of GMSCs and regulating the epithelial-to-mesenchymal transition during wound healing [43, 44], and Smad3 was identified as the downstream TGF-β1 effector [45, 46]. In addition, TGF-β1 signaling pathway plays an important role in fibrosis, briefly, TGF-β1 binding to TGF-β receptor leads to the phosphorylation of Smad2 and Smad3, and phosphorylated Smad2 and Smad3 subsequently form a complex, which translocates to the nucleus and interacts with nuclear transcription factors, where they regulate the transcription of specific fibrosis-related genes [41]. Hence, it could conceivably be hypothesized that under the microenvironment of BRONJ, the retarded gingival healing and impaired collagen deposition may be linked to suppressed TGF-β1 signaling pathway in BRONJ GMSCs.

For the underlying mechanism of BRONJ, based on our results, we infer that the dysfunction of BRONJ GMSCs and the suppressed TGF-β1 signaling pathway may be the pivotal factors in impaired gingival healing, ultimately leading to the occurrence of BRONJ. How do GMSCs dysfunction versus the suppressed TGF-β1 play the roles in impaired wound healing? It is well-known that wound healing is a natural physiological process restoring the function and integrity of damaged oral mucosa, which consists of four phases occurring in proper time and order. This process starts from hemostasis, then inflammation followed by proliferation and eventually tissue remodeling [47]. The sequence of events during wound healing is strictly programmed and any disturbances may impair normal wound healing. During the proliferative phase, GMSCs proliferate, adhere and migrate closing the wound, in which the dysfunction of BRONJ GMSCs plays a major role in the retarded wound healing of BRONJ patients. Moreover, it has been reported that TGF-β1 regulates the differentiation of GMSCs to fibroblasts, collagen synthesis in fibroblasts, matrix production by fibroblasts, and is considered to play a prominent role in wound healing [48]. In tissue remodeling phase, sequential and coordinated events including collagen synthesis followed by formation of granulation tissue, matrix degradation followed by replacement of collagen were largely regulated by TGF-β1, in which the suppressed TGF-β1 exerts the key role in impaired wound healing of BRONJ patients.

So far, the focus in BRONJ research has been on the bone—an “inside-out” theory [36, 49–52], it is equally important to speculate that the dysfunction of BRONJ GMSCs may play a critical role in the initiation of BRONJ—an “outside-in” hypothesis [53, 54]. In this “outside-in” theory, several key points must be emphasized. First, cancer patients receive a ten-fold higher dosage of BPs than those with osteoporosis, under long-term BPs treatment, there is a direct cytotoxic effect on GMSCs by blood support as well as the BPs enriched underlying bone. Thus, the cellular functions of GMSCs in BRONJ patients must be impaired. Second, GMSCs dysfunction and the suppressed TGF-β1 signaling further have the negative influence on the re-epithelialization and the collagen deposition, leading to delayed gingival healing. Third, retarded gingival healing led to the invasion of bacteria into the wound, contributing to inflammatory infiltration and the development of BRONJ.

## Conclusions

In summary, our results have demonstrated that under the microenvironment of oral mucosa at the site of BRONJ patients’ lesions, even at the recommended boundary of debridement, GMSCs exhibited decreased proliferation, adhesion, migration capacities and underwent early apoptosis in vitro. Furthermore, BRONJ GMSCs transplantation in a mice excisional skin model consistently exhibited poor wound healing effects. Mechanistically, we found the downregulation of TGF-β1 signaling both in BRONJ patients’ gingiva and BRONJ GMSCs transplantation animal model. But the limitation of the current study is that it is not known whether upregulating TGF-β1 signaling can promote soft tissue wound healing in preventing the onset of BRONJ. At the present time there are no available regimens for the prevention of BRONJ. Therefore, future studies will investigate therapeutic methods in improving the functions of BRONJ GMSCs and unravel the functional role of TGF-β1 signaling in BRONJ development.

## Supplementary Information

Below is the link to the electronic supplementary material.

Supplementary file1 (DOCX 95 KB)

## Data Availability

All data included in this study are available upon request by contacting with the corresponding author.

## Abbreviations

BRONJ: Bisphosphonate-related osteonecrosis of the jaw BRONJ
BPs: Bisphosphonates
GMSCs: Gingival mesenchymal stem cells
GFP: Green fluorescent proteins
RT-PCR: Real-time polymerase chain reaction
COLIA1: Collagen type I A1
TGF-β: Transforming growth factor beta
GO: Gene ontology
KEGG: Kyoto encyclopedia of genes and genomes
PBS: Phosphate buffered saline
MOI: Multiplicity of infection
H&E: Hematoxylin and eosin
DAPI: 40,6-Diamidino-2-phenylindole
TUNEL: Terminal deoxynucleotidyl transferase-mediated dUTP-biotin nick end labeling assay
TRITC: Tetramethyl Rhodamine Isothiocynate
IH: Immunohistochemistry
IF: Immunofluorescence
