# Supplementary material for: Characterization of Mesenchymal Stem Cells Derived from Bisphosphonate-Related Osteonecrosis of the Jaw Patients’ Gingiva
Source: Stem Cell Rev Rep. 2021 Sep 22;18(1):378–94. doi: 10.1007/s12015-021-10241-8 (PMC8799576; doi:10.1007/s12015-021-10241-8)
Supplement: Supplementary file 1 — Supplementary file1 (DOCX 95 KB) [file 12015_2021_10241_MOESM1_ESM.docx]

**Supplement 1**

Patients’ characteristics

| No | Age | Gender | BPs  types | BPs  doges | BPs  duration | Neoplasm histotypes | BRONJ duration | Group |
| --- | --- | --- | --- | --- | --- | --- | --- | --- |
| 1  2  3  4  5  6  7  8  9  10 | 57  68  54  74  81  52  65  51  56  51 | F  F  M  M  F  F  F  M  F  M | Zoledronic Acid  Zoledronic Acid Zoledronic Acid  Zoledronic Acid Zoledronic Acid  /  /  /  /  / | 4mg/M  4mg/M  4mg/M  4mg/M  4mg/M  /  /  /  /  / | 9M  12M  15M  24M  18M  /  /  /  /  / | Breast cancer  Renal cancer  Lung cancer  Breast cancer  Lung cancer  /  /  /  /  / | 3M  2.5M  3M  2.5M  1.5M  /  /  /  /  / | BRONJ  BRONJ  BRONJ  BRONJ  BRONJ  control  control  control  control  control |

**Supplement 2**

Total RNA was extracted using TRIZOL Reagent (Life technologies, Carlsbad, CA, US) following the manufacturer’s instructions and checked for a RIN number to inspect RNA integrity by an Agilent Bioanalyzer 2100 (Agilent technologies, Santa Clara, CA, US). Qualified total RNA was further purified by NucleoSpin RNA Clean-up XS kit (MN, Germany) and RNase-Free DNase Set (QIAGEN, GmBH, Germany). Total RNA were amplified, labeled and purified by using GeneChip 3' IVT PLUS Reagent Kit (Affymetrix, Santa Clara, CA, US) to obtain biotin labeled cRNA. Array hybridization and wash was performed using GeneChip® Hybridization, Wash and Stain Kit (Affymetrix, Santa Clara, CA, US) in Hybridization Oven 645 (Affymetrix, Santa Clara, CA, US) and Fluidics Station 450 (Affymetrix, Santa Clara, CA, US) followed the manufacturer’s instructions and then scanned using an GeneChip® Scanner 3000 (Affymetrix, Santa Clara, CA, US) and Command Console Software 4.0 (Affymetrix, Santa Clara, CA, US) with default settings.

**Supplement 3**

PCR primers for the specific amplification of human mRNA.

| Genes | Primers sequences | | |
| --- | --- | --- | --- |
| β-actin  COL1A1  TGF-β1  Smad2  Smad3  Smad4  α-SMA  FN | Forward  Reverse  Forward  Reverse  Forward  Reverse  Forward  Reverse  Forward  Reverse  Forward  Reverse  Forward  Reverse  Forward  Reverse | 5’-GGCACCACACCTTCTACAATGAGC-3’  5’-GATAGCACAGCCTGGATAGCAACG-3’  5’-GGCGAGAGAGGTGAACAAGG-3’  5’-GCCAAGGTCTCCAGGAACAC-3’  5’-AGCAACAATTCCTGGCGATACCTC-3’  5’-TCAACCACTGCCGCACAACTC-3’  5’-CTCTTCTGGCTCAGTCTGTTAA-3’  5’-AAGGAGTACTTGTTACCGTCTG-3’  5’-AGAGAGTAGAGACACCAGTTCT-3’  5’-GAAGTTAGTGTTTTCGGGGATG-3’  5’-ACAAGTAATGATGCCTGTCTGA-3’  5’-CTCCCATCCAATGTTCTCTGTA-3’  5’-TCGTGCTGGACTCTGGAGATGG-3’  5’-CCACGCTCAGTCAGGATCTTCATG-3’  5’-AATAGATGCAACGATCAGGACA-3’  5’-GCAGGTTTCCTCGATTATCCTT-3’ |  |
